# Pediatric MASLD in Severe Obesity: Current Clinical Perspectives on Diagnosis and Treatment

**DOI:** 10.3390/jcm15010137

**Published:** 2025-12-24

**Authors:** Ewa Kostrzeba, Mirosław Bik-Multanowski, Stephanie Brandt-Heunemann, Ewa Małecka-Tendera, Artur Mazur, Michael B. Ranke, Martin Wabitsch, Małgorzata Wójcik, Agnieszka Zachurzok, Elżbieta Petriczko

**Affiliations:** 1 Department of Pediatrics, Endocrinology and Diabetology of the Development Age, Pomeranian Medical University in Szczecin, 71-252 Szczecin, Poland; 2 Department of Medical Genetics, Jagiellonian University Medical College, 31-008 Cracow, Poland; 3 University Hospital, Institute of Human Genetics, LMU, 80336 München, Germany; 4 Division of Pediatric Endocrinology and Diabetes, Department of Pediatrics and Adolescent, Medicine, University Medical Center Ulm, 89075 Ulm, Germany; 5 German Center for Child and Adolescent Health (DZKJ), Partner Site Ulm, 89075 Ulm, Germany; 6 Department of Pediatrics and Pediatric Endocrinology, Medical University of Silesia, 40-752 Katowice, Poland; 7 Department of Pediatrics, Pediatric Endocrinology and Diabetes, Institute of Medical Sciences, Medical College of Rzeszów University, 35-301 Rzeszów, Poland; 8 Children’s Hospital in Tübingen, University of Tübingen, 72076 Tübingen, Germany; 9 Department of Pediatric and Adolescent Endocrinology, Pediatric Institute, Jagiellonian University Medical College, 31-008 Cracow, Poland; 10 Department of Pediatrics, Faculty of Medical Sciences, Medical University of Silesia in Zabrze, 41-800 Zabrze, Poland

**Keywords:** severe obesity, MASLD, childhood obesity

## Abstract

**Background:** Metabolic dysfunction-associated steatotic liver disease (MASLD) is now one of the most common chronic liver diseases in children and closely parallels the rising prevalence of severe pediatric obesity. **Methods:** This review synthesizes current evidence (2019–2025) and landmark studies on the diagnosis and management of pediatric MASLD, integrating contemporary guidelines and future directions. **Results:** Based on current data, we propose practical algorithms for screening and management of pediatric MASLD. Screening with alanine aminotransferase (ALT) as the initial test should be initiated in all children aged 9–11 years with obesity, in those who are overweight with additional risk factors, and in children with severe obesity or a family history of MASLD. Non-invasive imaging techniques show important limitations in children with increased amount of subcutaneous tissue requiring cautious interpretation. Lifestyle modification remains the cornerstone of therapy, while pharmacological treatments are investigational. In adolescents with severe obesity and significant comorbidities, bariatric surgery represents an effective therapeutic option with durable metabolic effects. **Conclusions:** Early identification of high-risk children, especially those with severe obesity, and implementation of multidisciplinary management are essential to prevent MASLD progression. Refinement of screening strategies and development of validated non-invasive biomarkers remain key priorities for future pediatric care.

## 1. Introduction

Over the past few years, metabolic dysfunction-associated steatotic liver disease (MASLD) has emerged as an increasingly significant pediatric health concern, paralleling the global rise in childhood obesity.

In 2020, an international expert panel redefined fatty liver disease related to metabolic dysfunction and introduced the term metabolic-associated fatty liver disease (MAFLD) as a replacement for non-alcoholic fatty liver disease (NAFLD) [1]. In 2023, four major international hepatology societies released a joint consensus introducing a new official terminology: MASLD (metabolic dysfunction-associated steatotic liver disease). This term replaces the previous NAFLD and MAFLD, emphasizing the metabolic basis of the disease and removing the stigmatizing reference to alcohol and the pejorative “fatty liver”. The recent shift from MAFLD to MASLD reflects an effort to unify the nomenclature of steatotic liver diseases across age groups. This change has particular implications, as it emphasizes the role of metabolic dysfunction while taking into account existing diagnostic pathways and epidemiological data derived from MAFLD-based criteria [2,3,4].

A recent meta-analysis including over 78 million individuals from 38 countries estimated the global prevalence of MASLD in children at 14.3%, while in children with obesity at 38.0% [5]. Among children with severe obesity—defined as a BMI-for-age greater than +3 z-scores according to WHO growth standards—it may reach up to 59% [6]. Significant geographical variations are observed, with the highest prevalence in North America (43.6%) and the lowest in Africa (31.1%) [7]. Global trend analyses indicate that the prevalence of pediatric MASLD nearly doubled from 4.6% in 2000 to 9.0% in 2017, with epidemiological projections suggesting it may reach approximately 30.7% by 2040 [8].

MASLD is driven by the interaction of metabolic, genetic, hormonal, and environmental influences. The major established risk factors include obesity, insulin resistance, and other components of metabolic syndrome such as increased waist circumference and dyslipidemia [9]. Male sex has been identified as an independent risk factor; it affects approximately 50% of boys and 35% of girls with obesity [10]. The marked increase in MASLD prevalence during puberty is closely linked to physiological insulin resistance, which promotes hepatic de novo lipogenesis and reduces fatty acid oxidation, facilitating lipid accumulation in hepatocytes [11]. Prenatal factors play a pivotal role in early disease programming. Maternal obesity and gestational diabetes induce long-term metabolic changes in the offspring through epigenetic reprogramming and altered placental nutrient transfer. Intrauterine growth restriction (IUGR) may further increase susceptibility to MASLD by promoting postnatal catch-up growth and reduced mitochondrial oxidative capacity [12]. In pediatric populations, genetic variants in PNPLA3 and TM6SF2 have been consistently associated with increased hepatic fat accumulation, elevated alanine aminotransferase (ALT) levels, and greater histological severity of MASLD, with additive effects observed when risk alleles co-occur [13,14]. A prospective study reported that first-degree relatives of patients diagnosed with MASLD and advanced fibrosis exhibit a markedly elevated risk of developing advanced fibrosis, highlighting the importance of implementing regular screening among these relatives [15]. Postnatal factors contributing to MASLD include the consumption of energy-dense, sugar-rich, and ultra-processed foods. Diets high in saturated fats and refined carbohydrates promote hepatic de novo lipogenesis, oxidative stress, and insulin resistance [16]. In contrast, inadequate intake of dietary fiber, omega-3 fatty acids, and antioxidant-containing foods further aggravates metabolic imbalance [17]. Additionally, sedentary behaviors and prolonged screen time decrease energy expenditure and foster insulin resistance, thereby intensifying hepatic fat accumulation [18].

**Figure 1:**
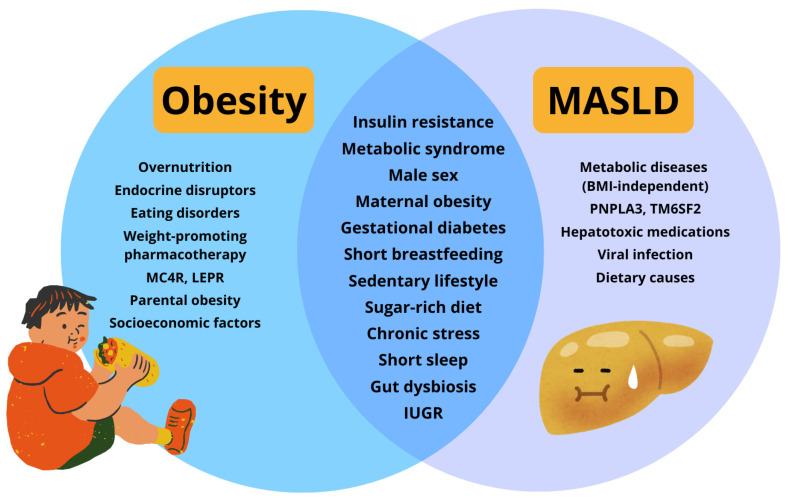
Interrelation of risk factors for MASLD development, with obesity as the major determinant. Abbreviations: intrauterine growth restriction (IUGR); body mass index (BMI).

Alarmingly, MASLD has been identified even in very young populations [19]. Among children aged 2–6 years, 21.5% were found to meet the diagnostic criteria for MASLD [20]. This phenomenon can be connected with the increasing incidence of early-onset severe obesity. A retrospective analysis of children with severe obesity in Poland showed that 83% of children had already met the criteria for severe obesity at their first recorded BMI assessment, with a median age of onset of 3.2 years [21]. The risk of MASLD increases sharply with rising body mass index, and in children with extreme obesity, profound metabolic derangements often manifest early and with remarkable intensity [22,23]. These disturbances accelerate hepatic fat accumulation and drive the transition from simple steatosis to steatohepatitis, fibrosis, and cirrhosis. Expansion of visceral adipose tissue, which is metabolically active and secretes proinflammatory adipokines such as TNF-α, IL-6, and leptin, intensifies hepatocellular injury [24]. Collectively, these metabolic, inflammatory, genetic, and behavioral mechanisms explain the accelerated course and greater severity of MASLD observed in children with severe obesity [25]. Figure 1 illustrates the interrelationships among the risk factors contributing to the development of MASLD, highlighting obesity as the predominant risk factor.

To date, no comprehensive review has specifically addressed pediatric MASLD in the context of severe obesity. This narrative review aims to synthesize current evidence on the diagnostic criteria, and therapeutic approaches relevant to MASLD in children with severe obesity.

## 2. Materials and Methods

A narrative literature review was conducted using the PubMed, Embase, and Cochrane Library databases. Search terms included “MASLD” OR “MAFLD” OR “NAFLD” AND “pediatric” OR “children” OR “adolescent” AND “obesity” OR “severe obesity” OR “morbid obesity” AND “risk factor” OR “diagnosis” OR “prophylaxis” OR “treatment” OR “management.” The review focused on studies involving pediatric populations, defined as children and adolescents up to 18 years of age. Studies conducted exclusively in adults were excluded unless they provided relevant pathophysiological or translational insights. Publications from 2019 to 2025 were prioritized to reflect the most recent evidence, evolving terminology, and updated clinical guidelines, and were complemented by selected landmark earlier studies of particular clinical or scientific importance. Study selection was narrative and based on clinical relevance, with an emphasis on meta-analyses, randomized controlled trials, and systematic reviews. Although the primary focus was on severe obesity, studies involving children with overweight and moderate obesity were also included to provide contextual comparison. Artificial intelligence tools were used to support language editing and improve clarity of the manuscript.

## 3. Results

### 3.1. Diagnosis

#### 3.1.1. Current Guidelines

MASLD is usually asymptomatic in children and is detected incidentally during the evaluation of obesity. Clinical manifestations, when present, are nonspecific and may include hepatomegaly or mild right upper quadrant discomfort [26].

Diagnostic criteria for MASLD in children and adolescents were established in 2021 [27]. According to the new definition, MASLD is diagnosed when there is evidence of hepatic fat accumulation—demonstrated by histology, imaging, or biochemical markers in combination with at least one of the following three conditions: excess adiposity (overweight or obesity), prediabetes or type 2 diabetes, or metabolic dysregulation. Metabolic dysregulation is defined as the presence of at least two age- and sex-specific metabolic risk factors, such as increased waist circumference, elevated blood pressure, hypertriglyceridemia, low high-density lipoprotein (HDL) cholesterol, impaired fasting glucose, or a triglyceride-to-HDL cholesterol ratio greater than 2.25, which serves as an indicator of insulin resistance [27].

Alanine aminotransferase (ALT) is currently recommended as the preferred screening test for pediatric MASLD, using sex-specific upper limits of normal (22 U/L for girls and 26 U/L for boys). It is recommended that all overweight children with a family history of MASLD (or other established risk factors) or obese children aged 9 years or older (BMI above the 95th percentile) undergo screening for MASLD. In multivariable analysis, children with class II and class III obesity had 2.1-fold (95% CI: 1.27–3.72) and 4.0-fold (95% CI: 2.41–6.96) greater odds of abnormal ALT, respectively, compared with those with class I obesity [28]. Persistent ALT elevation for more than three months above these thresholds should prompt further evaluation for MASLD and other chronic liver diseases [29,30].

Most clinical guidelines recommend performing abdominal ultrasound in children who show elevated ALT levels during screening [29,30,31]. Typical ultrasonographic features suggesting hepatic steatosis include increased hepatic echogenicity, reduced visualization of intrahepatic vessels, liver parenchyma, and diaphragm, as well as a hypoechoic renal cortex relative to the liver. However, the accuracy and interpretation of ultrasound findings can be significantly reduced in young children and in individuals with severe obesity due to excessive subcutaneous fat thickness. Another limitation of this method is that it can detect hepatic steatosis only when more than 30% of hepatocytes are affected.

Among non-invasive techniques, magnetic resonance imaging–proton density fat fraction (MRI-PDFF) demonstrated the best diagnostic performance for hepatic steatosis, while transient elastography with controlled attenuation parameter (TE-CAP) ranked second in accuracy [32,33,34]. Despite MRI-PDFF advantages, high cost and limited availability restrict their widespread use in clinical practice. Transient elastography studies indicate that, in children with MASLD, mild fibrosis occurs in about 66% of cases, significant fibrosis in 32%, advanced fibrosis in 15%, and cirrhosis in around 1% [33]. Hence, radiological assessment of pediatric patients should include the assessment of both steatosis and fibrosis [35]. So far, no strict pediatric cut-off values for assessing liver fibrosis by elastography have been established, however, given the considerable potential of these techniques, numerous ongoing studies aim to define and validate pediatric-specific thresholds [36,37].

Non-invasive diagnostic tools play a key role in the assessment of MASLD in children; however, their applicability is limited in children with severe obesity. Standard ultrasound has reduced sensitivity for detecting mild steatosis and is highly operator-dependent, with image quality and diagnostic precision compromised by increased subcutaneous tissue thickness. Similarly, transient elastography may be influenced by technical limitations giving unreliable measurements, and reduced reproducibility. These constraints may lead to underestimation or incorrect classification of steatosis and fibrosis severity, potentially delaying diagnosis, masking disease progression, or affecting referral and follow-up strategies. Consequently, results from non-invasive assessments should be interpreted with caution in children with severe obesity and integrated with clinical, biochemical, and longitudinal data when making management decisions.

Liver biopsy remains the gold standard for definitive diagnosis. Nevertheless, due to its invasive nature and sampling variability, biopsy is reserved for selected patients where histologic confirmation may alter management or prognosis [29].

Non-invasive fibrosis indices such as the Pediatric NAFLD Fibrosis Index (PNFI), Fibrosis 4 score (FIB-4), AST/Platelet Ratio (APRI), and Pediatric NAFLD Fibrosis Score (PNFS) have been evaluated in children, but their accuracy is limited and pediatric-specific cut-offs remain uncertain [38]. Comprehensive metabolic assessment is an integral part of the diagnostic process. Evaluation of fasting glucose, insulin, lipid profile, and glycated hemoglobin (HbA1c) helps identify cardiometabolic risk [29].

**Figure 2:**
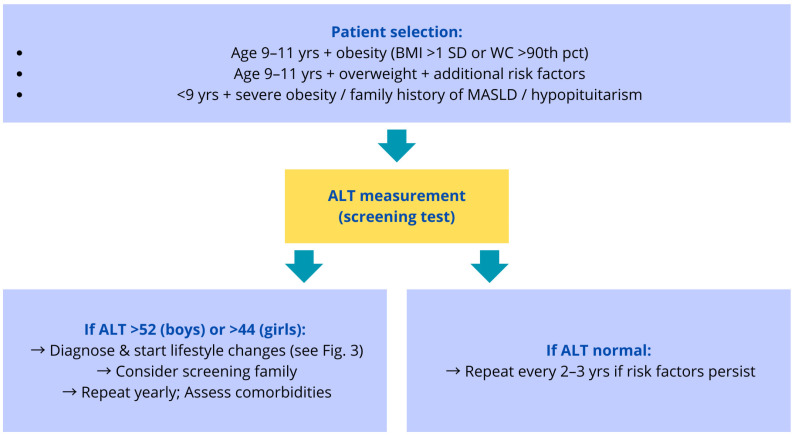
Proposed clinical screening algorithm for a child at risk of hepatic steatosis, based on current recommendations from major scientific societies [27,29,30,31]. Abbreviations explanation: body mass index (BMI), standard deviation (SD), waist circumference (WC), alanine aminotransferase (ALT), percentile (pct).

**Figure 3:**
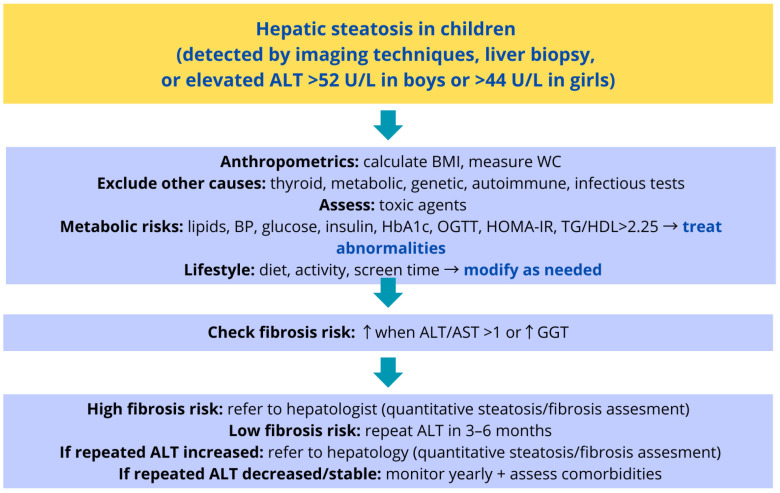
Proposed clinical management algorithm for a child diagnosed with hepatic steatosis, based on current recommendations from major scientific societies [27,29,30,31]. Abbreviations explanation: alanine aminotransferase (ALT), body mass index (BMI), waist circumference (WC) blood pressure (BP), glycated hemoglobin (HbA1c), oral glucose tolerance test (OGTT), homeostasis model assessment of insulin resistance (HOMA-IR), triglyceride-to-high-density lipoprotein cholesterol ratio (TG/HDL-C), aspartate aminotransferase (AST), gamma-glutamyl transferase (GGT).

Figure 2 presents a proposed algorithm for screening MASLD in children. The algorithm summarizes current recommendations from international expert groups and scientific societies [27,29,30,31] and provides a framework for identifying children who should undergo further evaluation. The subsequent steps of diagnostic work-up and management for children with confirmed hepatic steatosis are illustrated in Figure 3.

#### 3.1.2. Future Directions

The increasing prevalence of severe obesity among progressively younger children, along with the observation of a more aggressive course of disease has highlighted the need for novel noninvasive biomarkers [39,40]. This trend has become evident in a growing number of studies published between 2019 and 2025.

Current research points to associations between MASLD and elevated levels of uric acid, soluble CD163 (sCD163), interleukin-1 receptor antagonist (IL-1RA), osteopontin or osteonectin [41,42,43,44]. Moreover, circulating microRNA-660-5p (miR-660-5p) has been proposed as a potential biomarker for the presence of MASLD in preadolescent children, while miR-320a, miR-142-3p, miR-190a-5p, miR-374a-5p, and members of the let-7 microRNA family may serve as potential indicators of insulin resistance (IR) [45]. Expanding on these findings, recent metabolomic studies have identified characteristic abnormalities in amino acid profiles among overweight and obese prepubertal children, including elevated concentrations of branched-chain amino acids (BCAAs: isoleucine, leucine, valine) and aromatic amino acids (AAAs: tyrosine, phenylalanine), accompanied by increased alanine aminotransferase (ALT) and uric acid (UA) levels [46]. Together, these findings highlight a complex metabolic and inflammatory network underlying MASLD in children, suggesting that specific biochemical and molecular signatures may serve as early indicators of disease development and progression.

In adult populations, combining the triglyceride–glucose index (TyG index) with anthropometric measures such as waist circumference (WC) or BMI increases its utility as a noninvasive predictor of MASLD. In a cross-sectional study of 12,757 Korean adults, Song et al. demonstrated that the modified index TyG–WC (TyG = ln(fasting triglycerides (mg/dL) × fasting glucose (mg/dL))/2, TyG–WC = TyG × WC) achieved strong discriminative power for MASLD (AUROC ≈ 0.848) and yielded higher odds ratios for disease presence [47]. This association was further confirmed in the pediatric population. A recent cross-sectional analysis based on data from the 2017–2020 National Health and Nutrition Examination Survey (NHANES), which included 532 participants aged 12–18 years, demonstrated that TyG indices incorporating anthropometric parameters—particularly waist circumference (TyG–WC)—showed significantly stronger associations with hepatic steatosis assessed by the controlled attenuation parameter (CAP) [48]. These findings suggest that TyG–WC may serve as a simple, noninvasive, and clinically useful tool for early detection and risk stratification of MASLD in the pediatric population.

Lipid-derived indices have also attracted attention as simple, cost-effective tools for identifying MASLD in obese youth. The triglyceride-to-high-density lipoprotein cholesterol ratio (TG/HDL–C) has been proposed as a surrogate marker of hepatic steatosis and insulin resistance. In a 2024 cross-sectional study including 264 obese children, Trochimczyk et al. reported that TG/HDL–C demonstrated comparable diagnostic performance to the TyG index for MASLD detection (AUC = 0.638 vs. 0.641, respectively), with an optimal cut-off of approximately 2.5 (sensitivity 48.6%, specificity 76.3%) [49]. TG/HDL–C requires only a standard lipid profile, making it a potential practical first-line screening tool in routine pediatric practice, particularly where more advanced testing is unavailable.

Although these studies mainly have been conducted in relatively small pediatric cohorts and need further elaboration, they emphasize a current shift in diagnostic strategies toward the identification of new, noninvasive methods for detecting MASLD. Collectively, these data indicate that, after appropriate pediatric validation, noninvasive metabolic indices may become clinically relevant tools for MASLD screening and individualized management in children and adolescents.

### 3.2. Treatment

#### 3.2.1. Current Guidelines

Although several comprehensive clinical practice guidelines exist for the management of MASLD in adults, pediatric recommendations remain limited and less standardized.

The cornerstone of treatment for pediatric MASLD is lifestyle intervention, encompassing adherence to a healthy, well-balanced diet, engagement in daily moderate-to-vigorous physical activity, avoidance of sugar-sweetened beverages, and limiting screen time to less than two hours per day [29]. Despite these general principles, the current American Association for the Study of Liver Diseases (AASLD) recommendations emphasize that the optimal type of diet, as well as the intensity and duration of physical activity associated with the best therapeutic outcomes in children with obesity and MASLD, remain not fully elucidated [30]. Recent randomized intervention study showed that 12 weeks of high-intensity interval training significantly decreased ALT and AST levels and improved insulin sensitivity in overweight and obese adolescent girls highlighting that it could be an effective exercise therapy to prevent and reverse MASLD in adolescents with obesity [50]. A systematic review of thirteen articles indicated that adherence to a Mediterranean diet and lifestyle significantly reduces MASLD risk and improves metabolic parameters [51]. Family-based behavioral interventions should be supported as a key element in the management of pediatric MASLD. Several studies show that involving parents in behavioral programs leads to greater reductions in BMI z-score and improvements in diet, activity, and screen time compared with child-only approaches [52]. Moreover, a recent randomized trial in primary care demonstrated significant long-term weight improvements in children receiving family-based treatment [53]. Effective management of MASLD requires a multidisciplinary approach involving pediatricians, hepatologists, dietitians, psychologists, and physical activity specialists to ensure sustained adherence and address the complex metabolic and psychosocial aspects of the disease.

No pharmacotherapies are currently approved for treating MASLD in children [54]. No strong evidence supported the use of pharmacological treatment, including metformin was found. In the randomized, placebo-controlled trial of children with biopsy-confirmed MASLD, neither vitamin E nor metformin achieved a sustained reduction in ALT compared with placebo after 96 weeks of treatment [55]. The only form of pharmacologic therapy currently recommended is the treatment of comorbidities such as arterial hypertension, dyslipidemia, and type 2 diabetes. In children with MASLD and dyslipidemia, statins may be initiated from age 10 years when low-density lipoprotein (LDL) concentration remains ≥ 160 mg/dL, or ≥130 mg/dL with additional risk factors after 6–12 months of lifestyle intervention; omega-3 fatty acids can be considered for persistent hypertriglyceridemia (>200 mg/dL), while increased physical activity and dietary fiber support HDL-C improvement [39]. For those with comorbid type 2 diabetes, metformin remains the first-line pharmacologic therapy, improving insulin sensitivity and glycemic control, while GLP-1 receptor agonists are approved for children aged ≥ 10 years. Antihypertensive agents such as ACE inhibitors, ARBs, calcium channel blockers, or thiazide diuretics may be used when lifestyle modification fails to control blood pressure [56].

Current evidence indicates that bariatric surgery can reduce hepatic steatosis, inflammation, and fibrosis in patients with non-alcoholic steatohepatitis (NASH). Among available procedures, Roux-en-Y gastric bypass and vertical sleeve gastrectomy are both commonly regarded as safe and effective and performed in the pediatric age group [57]. Criteria for performing pediatric bariatric surgery include class 2 obesity (BMI ≥ 35 kg/m^2^ or ≥120% of the 95th percentile for age and sex, whichever is lower) accompanied by clinically significant comorbidities, such as type 2 diabetes, idiopathic intracranial hypertension, nonalcoholic steatohepatitis, Blount disease, slipped capital femoral epiphysis, gastroesophageal reflux disease, obstructive sleep apnea, cardiovascular risk factors (hypertension, dyslipidemia, insulin resistance), or markedly impaired health-related quality of life. Alternatively, surgery may be considered in adolescents with class 3 obesity (BMI ≥ 40 kg/m^2^ or ≥140% of the 95th percentile for age and sex, whichever is lower), regardless of the presence of comorbidities. Age alone is not a criterion for bariatric treatment eligibility [39]. However, research data in children under 12 years of age remains limited. Available evidence indicates that adolescents undergoing bariatric surgery experience persistent reductions in BMI, accompanied by significant improvement or remission of obesity-related comorbidities, including hypertension, type 2 diabetes, dyslipidemia, and other cardiovascular risk factors, as well as marked improvement in quality of life [58]. Most postoperative complications are minor, occur in the early postoperative period, and are primarily related to nausea and vomiting, affecting approximately 15% of patients [59]. Long-term risks include the development of micronutrient deficiencies, while 13–25% of patients may require additional surgical or endoscopic procedures within five years following the initial operation [60,61]. Study evaluating 10-year outcomes following pediatric bariatric surgery shown that in adolescents undergoing gastric bypass or sleeve gastrectomy at a mean age of 17 years, bariatric surgery resulted in an approximately 20% reduction in BMI, with comparable effectiveness between procedures. Long-term remission rates of type 2 diabetes, hypertension, and dyslipidemia exceeded 50%, especially with diabetes remission markedly higher than that reported in adults, supporting the indication for earlier surgical intervention [62].

**Figure 4:**
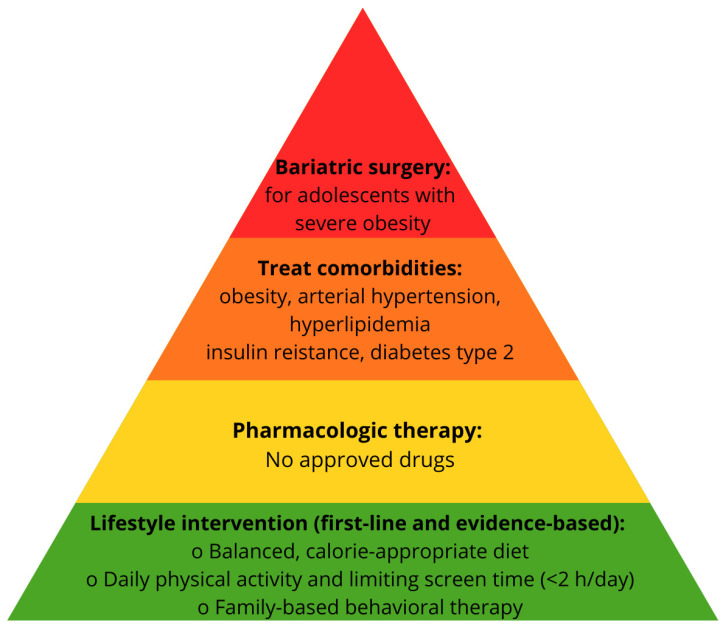
Algorithm for the management of pediatric MASLD.

A summary of current clinical practice guidelines for the management of pediatric MASLD is presented in Figure 4.

#### 3.2.2. Future Directions

A review of the literature from 2019 to 2025 has identified several promising directions for future research. A randomized controlled trial involving 53 participants reported that orlistat improved NAFLD-related and metabolic syndrome–related parameters compared with placebo after 12 weeks of treatment [63]. However, due to side-effects such as abdominal discomfort, bloating, gaseousness, diarrhea, fecal leakage and steatorrhea, orlistat is not well tolerated and therefore not commonly used in patients with obesity. Another study of 60 participants demonstrated that supplementation with 30 mg/day of elemental zinc for 16 weeks had a favorable effect on serum ALT, HDL-cholesterol, and inflammatory markers in overweight or obese children with NASH [64]. In addition to these findings, a pilot double-blind, placebo-controlled trial investigated N-acetylcysteine (NAC) therapy in children with obesity and biopsy-confirmed MASLD, showing significant improvements in oxidative stress, inflammation, and insulin resistance (IR), along with reductions in liver enzymes, fat fraction, and stiffness [65]. Combining pharmacotherapy with lifestyle modification appears to offer even greater therapeutic potential. In the meta-analysis by Omaña-Guzmán et al., which included 31 randomized clinical trials involving pediatric patients with MASLD and obesity, combined interventions of lifestyle modification and supplementation significantly reduced aminotransferase levels, insulin resistance indices, and BMI, although the overall clinical effect was moderate. Supplementation with vitamin D, probiotics, and omega-3 fatty acids (DHA) demonstrated potential hepatoprotective properties [66]. Similarly, a 12-week randomized trial in obese adolescents with MASLD showed that combining L-citrulline supplementation with high-intensity interval training led to significant improvements in liver enzymes, insulin resistance, and arterial stiffness compared with either intervention alone [67]. Recent phase 3 data with the glucagon-like peptide-1 receptor agonist semaglutide in adults with NASH and fibrosis have demonstrated substantial improvements in steatohepatitis and weight reduction, highlighting the potential of incretin-based therapies in this field. However, dedicated pediatric trials and long-term safety data are essential before such treatments can be recommended in children [68].

Recently, increasing evidence from preclinical and clinical studies indicates that individuals with MASLD exhibit heightened sympathetic nervous system activity, which may amplify key mechanisms underlying hepatic steatosis, insulin resistance, and metabolic dysregulation. Sympathetic overdrive has also been linked to disease progression and fibrosis development in adult MASLD. These observations provide a rationale for targeting neurogenic pathways as a novel therapeutic strategy. In experimental high-fat diet models, renal and hepatic sympathetic denervation reduced hepatic insulin resistance, decreased hepatic glucose production, and reversed hepatic steatosis. Such findings support a potential mechanistic role of sympathetic modulation in metabolic liver disease. However, clinical data evaluating the metabolic effects of sympathetic denervation remain inconsistent [69]. Importantly, available trials were not specifically designed to assess MASLD-related outcomes. Evidence in pediatric populations is currently lacking. Therefore, future studies should explore the safety, feasibility, and efficacy of sympathetic modulation in carefully selected adolescents with severe obesity and high metabolic risk.

Assessing treatment response in pediatric MASLD remains challenging due to the lack of validated, age-appropriate noninvasive markers [54]. Although a reduction in ALT by 10 U/L over 96 weeks has been associated with a 1.28-fold relative increase in the odds of histologic improvement, ALT remains an imperfect surrogate [55]. Future research should prioritize the development of standardized noninvasive biomarkers and the implementation of well-designed, randomized multicenter trials using histologic or MRI-based endpoints to more accurately assess therapeutic efficacy and inform evidence-based guidelines for the management of pediatric MASLD.

## 4. Discussion

Pediatric MASLD in the setting of severe obesity is an increasingly prevalent challenge. Recent U.S. data (*n* = 25,847) show a 253% rise in extremely severe obesity between 2008 and 2023, particularly among older adolescents, strongly associated with MASLD, insulin resistance, dysglycemia, and metabolic syndrome [70]. This highlights the need for earlier prevention, risk stratification, and public health actions.

Children with severe obesity constitute the highest-risk group for MASLD, yet excessive adiposity can hinder accurate assessment. Standardization of non-invasive imaging (e.g., elastography) with validated pediatric cut-offs could substantially improve diagnostic accuracy in this population.

Given the correlation between obesity severity and liver disease progression, screening strategies should prioritize children with severe obesity. Current recommendations initiating screening at 9–11 years may be insufficient in the context of earlier-onset severe obesity, warranting re-evaluation of both timing and frequency of follow-up.

Additionally, the evolving terminology from MAFLD to MASLD underscores the need for consistent, universally accepted diagnostic definitions to facilitate research comparability and cohesive clinical practice. From clinical point of view, the transition from MAFLD to MASLD is only terminological and does not substantially alter current diagnostic strategies. However, adoption of the MASLD terminology support longitudinal follow-up of pediatric patients transitioning into adult care and improve comparability across studies.

Compared with existing pediatric guidelines the algorithms proposed in this review place greater emphasis on children with severe obesity as the highest-risk group for MASLD progression. Current recommendations provide general screening principles, but do not fully address the practical limitations of non-invasive diagnostic tools in children with extremely high BMI or the need for earlier and more intensive evaluation. By consolidating screening, diagnostic work-up, and management the proposed algorithms aim to improve clinical decision-making and facilitate care in routine pediatric practice.

Children with severe obesity represent a particularly vulnerable subgroup, with the greatest risk of advanced disease and the greatest diagnostic challenges. Efforts should focus on optimizing non-invasive diagnostics, refining screening protocols, and developing targeted therapeutic approaches. Ultimately, early identification and proactive management of extreme obesity in childhood may offer the best strategy to prevent MASLD progression and long-term metabolic consequences.

In summary, MASLD in children differs from adult disease due to ongoing growth and puberty, which limit the direct applicability of adult diagnostic and treatment criteria. For pediatricians, the key tasks are early identification of high-risk children, consistent use of validated screening tools, and contribution to well-designed pediatric clinical studies.

## Data Availability

No new data were created or analyzed in this study. Data sharing is not applicable to this article.

## Abbreviations

The following abbreviations are used in this manuscript:

AASLD	American Association for the Study of Liver Diseases
ACE	angiotensin-converting enzyme inhibitors
ALT	alanine aminotransferase
APRI	AST to Platelet Ratio Index
ARBs	angiotensin receptor blockers
AST	aspartate transaminase
BMI	body mass index
CAP	controlled attenuation parameter
FIB-4	Fibrosis-4 score
GLP-1	glucagon-like peptide-1 receptor agonists
HbA1c	glycated hemoglobin
HDL	high-density lipoprotein cholesterol
IL-1RA	interleukin-1 receptor antagonist
IR	insulin resistance
LDL	low-density lipoprotein
MAFLD	metabolic-associated fatty liver disease
MASLD	metabolic dysfunction–associated steatotic liver disease
MRI-PDFF	magnetic resonance imaging–proton density fat fraction
miR-660-5p	circulating microRNA-660-5p
NAC	N-acetylcysteine
NAFLD	non-alcoholic fatty liver disease
NHANES	National Health and Nutrition Examination Survey
NASH	non-alcoholic steatohepatitis
PNFI	Pediatric NAFLD Fibrosis Index
PNFS	Pediatric NAFLD Fibrosis Score
sCD163	soluble CD163
TE-CAP	transient elastography with controlled attenuation parameter
TG/HDL-C	triglyceride-to–high-density lipoprotein cholesterol ratio
TyG	triglyceride–glucose index
TyG-WC	triglyceride–glucose index adjusted for waist circumference
WC	waist circumference
